# Investigating the influencing factors of vaccination decisions for newly developed and established vaccines: a comparative study based on latent class logit models in China

**DOI:** 10.3389/fpubh.2024.1455718

**Published:** 2024-08-29

**Authors:** Shiyun Chang, Biao Xu, Hailing Xi, Yifan Shao

**Affiliations:** School of Government, Nanjing University, Nanjing, China

**Keywords:** COVID-19 vaccines, influenza vaccines, latent class logit model, choice experiment, vaccine preferences

## Abstract

**Background:**

The factors influencing vaccination decision-making for newly developed vaccines may be similar to and different from those for established vaccines. Understanding these underlying differences and similarities is crucial for designing targeted measures to promote new vaccines against potential novel viruses.

**Objective:**

This study aims to compare public vaccination decisions for newly developed and established vaccines and to identify the differences and similarities in the influencing factors.

**Method:**

A discrete choice experiment (DCE) was conducted on 1,509 representatives of the general population in China to collect data on preferences for the coronavirus disease 2019 (COVID-19) and influenza vaccines, representing the newly developed and established vaccines, respectively. The latent class logit model was used to identify latent classes within the sample, allowing for an analysis of the factors distinctly influencing choices for both types of vaccines.

**Result:**

Participants valued similar attributes for both vaccines. However, concerns about sequelae were more significant for the newly developed vaccine, while effectiveness was prioritized for the established vaccine. Class membership analysis revealed these differences and similarities were significantly correlated with age, health, yearly household income, acquaintances’ vaccination status, and risk perception.

**Conclusion:**

The study highlights the need for tailored communication strategies and targeted vaccination interventions. For the newly developed vaccines, addressing concerns about side effects is more crucial. For long-standing vaccines, emphasizing their effectiveness can enhance uptake more significantly. Engaging healthcare providers and community influencers is essential for both vaccines to increase public confidence and vaccination rates. Clear communication and community engagement are critical strategies for addressing public concerns and misinformation, particularly during periods of heightened concern.

## Highlights

- We identified the similarities and differences in factors influencing preferences for newly developed and established vaccines.
- Vaccine sequelae were prioritized for the newly developed vaccines.
- Vaccine effectiveness was regarded as most important for the established vaccines.
- Medical experts’ vaccination experiences influenced preferences for the new vaccines.
- Friends/relatives’ vaccination experiences impacted preferences for long-standing vaccines.

## 1 Introduction

Over the past decades, the world has experienced multiple infectious disease outbreaks, with seasonal influenza causing up to 650,000 deaths each year and COVID-19 causing over 7,000,000 deaths since its outbreak (1, 2). The world will continue to face serious virus threats, posed by variants of existing viruses but also novel diseases (3). Vaccination has emerged as a crucial strategy for preventing and controlling morbidity and mortality associated with viruses (4, 5). New vaccines not only reduce the incidence and severity of novel infections but also alleviate the burden on healthcare systems, thereby safeguarding public health and safety (6).

However, for new vaccines against emerging viruses, there is often a lack of extensive clinical trials and data supporting their credibility and safety (7). Compared to well-established vaccines, distrust and inadequate confidence in newly developed vaccines are more pronounced, especially during urgent epidemics (8). This can be exemplified by the influenza and COVID-19 vaccines. Influenza vaccines, with their long history of use, are generally seen as safer. In contrast, during the COVID-19 pandemic, the rapid development and deployment of “rush” vaccines under emergency use authorizations led to more public skepticism and concerns about their efficacy and safety, as well as the credibility of pharmaceutical companies (4, 9, 10).

As a result, despite the availability of new vaccines, vaccine hesitancy is more prevalent than existing vaccines during emerging pandemics (8). Vaccine hesitancy refers to delay or refusal of vaccination despite the availability of vaccines (5). In other words, individuals exhibit varying decision-making processes and preferences when confronted with newly developed or established vaccines, each with its different characteristics. To address a potentially large-scale virus outbreak in the future, it is insufficient to solely depend on the previous old vaccine data. Understanding public decisions for both types of vaccines and how various factors influence these decisions is crucial for enhancing new vaccine uptake and designing effective public health strategies for potential novel outbreaks in the future. In recent years, there have been numerous studies on vaccines for long-standing viruses, alongside an increasing focus on vaccines for newly emerged viruses, particularly since the COVID-19 pandemic (11, 12). Previous studies have provided preliminary data suggesting a strong overlap among various concerns related to diverse types of vaccines (13). In addition to vaccine attributes, public preferences are also influenced by non-attribute factors.

All influential factors can be summarized into:

(1) Vaccine characteristics: Individuals primarily prefer vaccines with higher effectiveness (14–16), fewer side effects (17–20), and longer protection (19, 21, 22). Besides, they secondarily pay attention to vaccine source (23), costs (24, 25), number of doses (11), vaccination location, etc. (26–30).

(2) Individual characteristics: Most evidence has proven older adults (9, 20), males (31–33), or healthier individuals (32, 34) are more likely to accept vaccination. Some studies have indicated that individuals with lower levels of education or income express a greater willingness to get vaccinated (35, 36), while others present conflicting evidence (8, 37, 38). Additionally, in areas with significant ethnic diversity, ethnicity is also a contributing factor (9, 20, 39). Moreover, when vaccination decisions involve kids, individuals exhibit more concerns about vaccines, making parental status another influencing factor (40–42).

(3) Social support: The behaviors of family, friends, authority, and healthcare providers make a difference in individuals’ vaccination decision-making (43, 44). Political movements, recommendations from trusted healthcare professionals, and positive endorsements from social networks can enhance vaccine acceptance (12, 44–46). Shared information and news coverage in media can change public attitudes toward vaccination (26, 44, 47). The government also plays an essential role in public vaccination decisions (21). For example, if one specific vaccine was free for the public or was mandated by the government, individual vaccination perceptions and concerns could be greatly changed.

(4) Psychological factors: Individuals tend to experience fear, anxiety, and mistrust when faced with uncertainty and risk (12), which can be attributed to the risk perception affecting the decision-making process. Risk perception toward injection and vaccines is pivotal to vaccination decision-making, mainly including perceived susceptibility to injection, perceived severity of injection, perceived susceptibility to vaccine side effects and perceived severity of side effects (8, 33, 44, 48–51). Additionally, individuals can perceive benefits from and barriers to vaccination. For instance, perceived vaccine effectiveness and safety encourage individuals to get vaccinated; conversely, perceived cost, misinformation, or complex procedures deter individuals from getting vaccinated (41, 48, 52).

Nevertheless, most existing studies focus on the preferences for one specific vaccine. There is limited research comparing public preferences for new vaccines and old vaccines, particularly identifying the differences and similarities in the influencing factors. This study aims to fill the gap. Our study initially aims to elicit differences and similarities in public preferences for newly created and well-established vaccines. The second objective is to analyze the factors influencing different public preferences for both types of vaccines.

The COVID-19 vaccines provide the opportunity for this comparison. China, in order to respond quickly to the pandemic, expedited the deployment of vaccines, such as Sinovac (53). The COVID-19 vaccines were characterized by their accelerated development, limited testing, and urgent deployment, making them an ideal example of the newly created vaccine in our study. In contrast, seasonal influenza was chosen as the established vaccine because both influenza and COVID-19 are respiratory illnesses with similar modes of transmission. Additionally, the influenza vaccine has been in long-standing use, providing extensive data on its effectiveness in preventing influenza and reducing its severity, owing to its seasonal nature.

To our knowledge, our study is the first to explore differences and similarities in preferences for newly created and well-established vaccines based on DCEs. This study can provide insights into designing tailored communication and distribution strategies to enhance the uptake of established vaccines such as the influenza vaccine, as well as future novel vaccines developed for potential emerging virus outbreaks, thereby addressing vaccine hesitancy based on vaccine type.

## 2 Methods

### 2.1 Discrete choice experiments

DCEs were utilized to capture public preferences for vaccines. This quantitative method involves choice scenarios presenting respondents with a series of hypothetical vaccine profiles, each varying in specific vaccine characteristics, namely attributes and levels (e.g., attribute: effectiveness, safety, and costs of the vaccine; attribute levels: for the effectiveness of a vaccine: 40, 70, and 90%) (54). The DCE approach is a suitable method for eliciting preferences, effectively reflecting trade-offs that individuals are willing to make among different vaccine attributes (54). This method has been widely validated in health research, ensuring its reliability in understanding which attributes most influence vaccine choices (18, 55).

### 2.2 Choosing attributes and attribute levels

As the COVID-19 vaccines were chosen as the newly created vaccines, and the influenza vaccines as the established vaccines, the attributes and levels used in the DCE were carefully selected based on literature review, expert consultation, and preliminary qualitative research specific to these vaccine types.

First, by searching for literature review, eight important attributes were initially identified: effectiveness, duration of protection, vaccine source, out-of-pocket costs, vaccine sequelae, number of doses, vaccination location, and frequency of vaccination (11, 17–19, 21, 23–30, 56, 57). Next, we conducted interviews with six experts in the field of vaccination and four focus groups with the general population aged over 18 years old, which allowed us to select the final key attributes: vaccine effectiveness, protection duration, sequelae, origin, and out-of-pocket costs.

It is essential to ensure that the attribute levels cover a meaningful range and reflect realistic scenarios faced by individuals when making vaccine-related decisions. Therefore, for the effectiveness and protection duration attributes, their levels were categorized based on clinical trial data. For the sequelae following vaccination, sequelae are more commonly reported with the COVID-19 vaccines compared to the influenza vaccines, so this attribute was ranked into three levels for the COVID-19 vaccines and two for the influenza vaccines (15, 16, 22, 58–61). For the origin of vaccines, respondents can choose between imported and domestic options. For the cost attribute, while COVID-19 vaccines may be free of charge in many countries, including cost as an attribute can help understand individuals’ perceived value. Its levels were similarly based on real-world vaccine prices (62, 63). The descriptions of the attributes and attribute levels are summarized in Table 1.

**Table 1:** Attributes and levels in the DCEs.

Notation	Definition	Levels	
		COVID-19	Influenza
Effectiveness (%)	The degree to which vaccination reduces the risk of disease compared with the non-vaccinated	75 and 95%	40, 70 and 90%
Protection duration (year)	The durability of the vaccine effect	0.5,1, and 5	0.5 and 1
Sequelae	Probability of disability, hospitalization, life-threatening reaction or death following vaccination	Low, middle and high	Low and middle
Country-of-origin	Origin of product	Imported and domestic products	
Out-of-pocket cost (CNY)	Money individuals need to pay on their own	0, 150, and 300	

CNY, the Chinese Yuan.

### 2.3 Experimental design and questionnaire

The study design involved creating a questionnaire that included the DCE tasks and additional questions on other information. The questionnaire was pre-tested to ensure clarity and relevance. We selected 30 individuals from various demographic backgrounds, including different age groups, education levels, and socio-economic statuses. Participants completed the questionnaire under conditions similar to those of the actual survey, and we observed their responses to identify any issues with understanding or interpretation. After completing the questionnaire, participants provided feedback on confusing, ambiguous, or irrelevant questions, and suggested improvements for clarity and comprehension. We analyzed the feedback and refined the wording of questions, adjusted response options, and ensured alignment with the study’s objectives before implementation for data collection. Survey questions and information were designed to be clear and unbiased to minimize inaccuracies in reporting. Particularly, we have asked the participants in the pre-test if they viewed influenza vaccines as established and COVID-19 vaccines as newly developed. They consistently agreed with this statement. To further ensure the different opinions toward these vaccines, the timeline for each vaccine’s development and use was provided in the questionnaire. Additionally, we assured participants of the confidentiality and anonymity of their responses in the questionnaire to reduce social desirability bias.

The DCE tasks consisted of a series of choice sets where respondents were asked to select their preferred vaccine from pairs of hypothetical profiles. Each choice set presented vaccines with different combinations of the selected attributes and levels. A fractional factorial design was used to reduce the number of potential combinations to a manageable number while ensuring that the main effects and key interactions could be estimated. The D-efficient design was used to generate 40 choice tasks in STATA 17.0.

**Figure 1:**
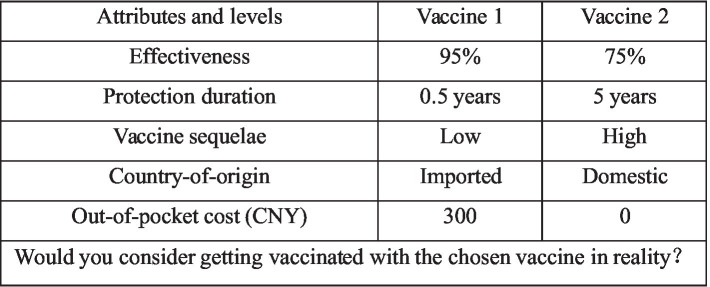
Example of choice sets.

To reduce the burden brought by excessive choice tasks, the 40 choice sets were randomly divided into 4 versions, with each version containing 10 choice sets. Each choice set consisted of two hypothetical alternatives, and respondents were asked to indicate their preference and whether they would choose to be vaccinated in reality. An example of one such choice task is shown in Figure 1. The procedure was consistently applied to both the COVID-19 vaccine group and the influenza vaccine group.

The other part of the questionnaire collected information on respondents’ background characteristics, vaccination status, and risk perception. Background characteristics comprised age, gender, educational level, health condition, place of residence, occupation, marital status, and yearly income. Vaccination status involved four questions asking whether respondents, their friends/relatives, and acquainted leaders and doctors have been vaccinated. Risk perception encompasses the assessment of both the severity of virus infection and potential adverse effects following receiving a vaccine, as well as the likelihood of contracting viruses and the potential adverse effects.

### 2.4 Data collection

The survey was conducted between October and December 2021 in China, a period shortly after the rollout of new COVID-19 vaccines and during the flu season.

Initially, a stratified random sampling method was used to select four provinces (Guangxi, Henan, Anhui, and Jiangsu), one autonomous region (Xinjiang), and one municipality (Shanghai), based on geographical location and GDP *per capita*. Following this, 20 cities were randomly chosen from the selected provinces and the autonomous region according to GDP *per capita*. Subsequently, two or three typical communities were selected from each city (or municipality) that had vaccination sites capable of providing vaccination services.

Next, to determine the required sample size, a power analysis was conducted using G*Power 3.1. For a medium effect size (d = 0.5), with an alpha level of 0.05 and power of 0.80, the analysis indicated that a minimum sample size of 64 participants for each type of vaccine was needed. Generally, a sample size of over 100 respondents is highly recognized and recommended for DCE studies (64). Considering the vast geographic scope and diverse population of China, as well as the study’s focus on sample heterogeneity, we decided to expand the sample size to 800 respondents for each vaccine. This would help better capture variability across different regions and population groups, enhancing the stability and accuracy of model estimates.

Then, the survey was promoted both online and offline within the communities to encourage participation among residents. Residents aged 18 years or older in each community were invited to participate in our survey. Respondents were randomized into two hypothetical vaccine scenarios (COVID-19 vaccine group or influenza vaccine group), and four versions of choice sets were evenly distributed within each group. In each version, 10 choice tasks were sequence-randomized to mitigate order bias. This methodology ensured a comprehensive and unbiased representation of preferences regarding vaccination in the target population.

We collected respondents’ preferences for the vaccines and additional individual information via Sojump, a web-based platform designed for conducting online surveys. We provided participants with the questionnaire link or quick response (QR) code. Before answering the questionnaire, key terminologies were explained and notes were provided to guide respondents to complete the survey. For those with difficulties in understanding the questions, our research team offered assistance, including one-on-one support and clarification of any confusing items, to ensure that all respondents could participate fully. This approach helped enhance the quality and reliability of the data collected. As a result, a sample of 1,509 adult respondents was recruited from 20 cities and 1 municipality, providing a representative sample of the population in these regions.

Ethical guidelines were strictly followed, and all participants provided informed consent prior to participating. The study has been approved by the Medical Ethics Sub-Committee of the Ethics Committee for Science and Technology at Nanjing University (Approval Number: OAP20230407001).

### 2.5 Statistical analysis

In analyzing the DCE data, we used two models: the conditional logit model (CLM) and the latent class model (LCM). The CLM was applied to focus on how vaccine attributes influence individual vaccine options. The LCM allowed for individual heterogeneity by incorporating individual-specific characteristics beyond just vaccine attributes, which is crucial for uncovering distinct population segments that may prioritize different vaccine attributes. This approach provides segment-specific insights, allowing for a more nuanced understanding of the factors influencing vaccine decisions among various subgroups, making it suitable for our study’s objectives.

All data analyses were conducted using Stata 17.0. The various indicators obtained from the analysis are categorized into two main types: (1) Preference indicators: Odds ratio (OR) indicate the direction and magnitude of attribute preferences. Regression coefficients explain whether socio-demographic characteristics influence preferences. Number of classes indicates the existence of preference heterogeneity. (2) Model fit indicators: likelihood ratio test statistics, pseudo-R^2^, Akaike Information Criterion (AIC), Consistent Akaike Information Criterion (CAIC), and Bayesian Information Criterion (BIC).

In the CLM, it is assumed that preferences are consistent across individuals. The probability of choosing an option i is given by Eq. (1):


(1) $$P_{i}=\frac{e^{v\left(\beta ,X_{i}\right)}}{\sum_{j}e^{v\left(\beta ,X_{j}\right)}}$$

where $v\left(\beta ,X_{i}\right)$ represents the utility function of option i , which is a linear combination of its attributes x and their corresponding coefficients β .

The LCM divided participants into several classes, each with its own set of preference regression coefficients, which implies homogeneity within classes and heterogeneity across classes. In other words, each class represents a CLM. The probability of selecting the option i for participants belonging to the class q is given by Eq. (2):


(2) $$P_{i}\left(\mathrm{class}=q\right)=\frac{e^{v\left(\beta_{q},X_{i}\right)}}{\sum_{j}e^{v\left(\beta_{q},X_{j}\right)}}$$

The LCM can also incorporate a set of covariates (such as demographic characteristics, vaccination behaviors of acquaintances, and risk perception) to classify the sample. The class membership probability is given by Eq. (3):


(3) $$P\left(\mathrm{class}=q\right)=\frac{e^{v\left(\alpha_{q},Z\right)}}{\sum_{q}e^{v\left(\alpha_{q},Z\right)}}$$

where *P* $\left(\mathrm{class}=q\right)$ is the probability that each individual belongs to class q given their characteristics Z and α are the coefficients for the covariates Z .

## 3 Results

### 3.1 Characteristics of respondents

The sample consists of 746 respondents for the COVID-19 vaccine group and 763 respondents for the influenza vaccine group. For both subgroups, the mean age of respondents was approximately 40 years old, and the age distribution was similar, with the majority falling into the age range of 18–24 and 25–34, followed by other age groups up to 65 and above. For the COVID-19 and influenza groups, slightly over half of the respondents were female (56.43 and 64.22%), married (54.96 and 58.72%), lived in rural regions (52.14 and 51.38%), and had a yearly income between 50,000 and 200,000 yuan (52.01 and 63.30%); and a significant proportion of respondents were students (32.04 and 39.45%), had a bachelor’s degree or above (59.92 and 65.14%), and reported good health or above (82.71 and 82.57%).

Although the percentages of respondents and their acquaintances who have been vaccinated were remarkably high in both groups, the proportions in the COVID-19 group were generally higher than those in the influenza group. While respondents perceived a higher severity of harm from COVID-19 infection compared to influenza infection (81.10% versus 64.22%), they perceived a lower-than-average probability of COVID-19 infection compared to influenza infection (58.10% versus 21.10%). Similarly, there were more perceptions of the severity of vaccine sequelae from COVID-19 vaccines than influenza vaccines (49.87% versus 47.71%), but respondents perceived a lower-than-average probability of experiencing COVID-19 vaccine sequelae compared to influenza vaccine (56.84% versus 26.61%) (Table 2).

**Table 2:** Participants’ demographic characteristics, vaccination, and risk perception.

Variables	COVID-19 vaccine (*N* = 746)		Influenza vaccine (*N* = 763)	
Demographic characteristics	*N*	%	*N*	%
Age (mean; std.dev)	40.03	19.16	40.04	24.43
*Age range*				
18–24	233	31.23%	294	38.53%
25–34	116	15.55%	84	11.01%
35–44	131	17.56%	84	11.01%
45–54	78	10.46%	63	8.26%
55–64	48	6.43%	84	11.01%
≥65	140	18.77%	154	20.18%
Gender				
Male	325	43.57%	273	35.78%
Female	421	56.43%	490	64.22%
*Marital status*				
Unmarried	309	41.42%	301	39.45%
Married	410	54.96%	448	58.72%
Others	27	3.62%	14	1.83%
*Education*				
Elementary school or below	84	11.26%	77	10.09%
Middle school	110	14.75%	84	11.01%
High school or technical secondary school	105	14.08%	105	13.76%
Bachelor degree	390	52.28%	2,205	57.80%
Master, PhD or above	57	7.64%	56	7.34%
*Health*				
Very good	344	46.11%	189	24.77%
Good	273	36.60%	441	57.80%
Moderate	111	14.88%	98	12.84%
Poor	13	1.74%	35	4.59%
Very poor	5	0.67%	0	0
*Place of residence*				
Urban	357	47.86%	371	48.62%
Rural	389	52.14%	392	51.38%
*Occupation*				
Student	239	32.04%	301	39.45%
Employee in private organization	169	22.65%	140	18.35
Public official	68	9.12%	91	11.93%
Others	270	36.19%	231	30.28%
*Yearly household income* (CNY)				
≤K	102	13.67%	98	12.84%
50–100 K	174	23.32%	217	28.44%
100–200 K	214	28.69%	266	34.86%
200–400 K	126	16.89%	126	16.51%
400–600 K	74	9.92%	49	6.42%
600–1,000 K	21	2.82%	7	0.92%
≥1000 K	35	4.69%	0	0
*Get vaccinated*				
Individuals	716	95.98%	80	10.48%
Acquainted doctors	598	80.16%	64	8.39%
Acquainted friends/relatives	705	94.50%	67	8.78%
Acquainted leaders	647	86.73%	79	10.35%
*Risk perception*				
Harm of infection				
Severe	605	81.10%	490	64.22%
Moderate	107	14.34%	203	26.61%
Mild	34	4.56%	70	9.17%
*Probability of infection*				
Higher than the average	191	25.60%	350	45.87%
Similar to the average	129	17.29%	252	33.03%
Lower than the average	426	58.10%	161	21.10%
*Harm of vaccine sequelae*				
Severe	372	49.87%	364	47.71%
Moderate	218	29.22%	329	43.12%
Mild	156	20.91%	70	9.17%
*Probability of vaccine sequelae*				
Higher than the average	173	23.19%	196	25.69%
Similar to the average	149	19.97%	364	47.71%
Lower than the average	424	56.84%	203	26.61%

### 3.2 CLM results

In both groups, five attributes were significant (*p < 0.1* for all attributes but *p < 0.05* for 5-year protection duration) and have expected signs, indicating that all attributes were important and respondents preferred domestic vaccines with more effectiveness, longer protection duration, lower probability of vaccine sequelae, and less out-of-pocket cost. In the COVID-19 vaccine group, the vaccine sequelae attribute emerged as the most influential factor, followed by the effectiveness attribute. The protection duration, origin, and out-of-pocket cost attributes appeared to have relatively less impact on individuals’ preferences. In the influenza vaccine group, the vaccine sequelae and effectiveness attributes were identified as the primary and secondary drivers of preferences, respectively, while the importance of the other three attributes was comparatively lower (Table 3).

**Table 3:** Conditional logit model of public preferences affected by vaccine attributes.

Attribute	COVID-19 vaccine (N = 746)		Influenza vaccine (N = 763)		
	OR	95% CI		OR	95% CI
Effectiveness (base: 75%)			(base:40%)		
95%	1.86^a^	1.72, 2.00	70%	1.37^a^	1.21, 1.54
			90%	3.65^a^	3.32, 4.02
Protection duration (base: 6 months)					
1 year	1.10^a^	1.00, 1.21	1 year	1.18^a^	1.09, 1.27
5 years	1.65^b^	1.50, 1.80			
Sequelae probability (base: high)			(base: middle)		
Middle	2.05^a^	1.85, 2.27	Low	1.86^a^	1.72, 2.01
Low	4.67^a^	4.23, 5.16			
Country-of-origin (base: imported products)					
Domestic products	1.50^a^	1.39, 1.62		1.24^a^	1.16, 1.34
Out-of-pocket cost (CNY) (base:0)					
150	0.65^a^	0.60, 0.70		0.85^a^	0.78, 0.93
300	0.46^a^	0.41, 0.50		0.42^a^	0.37, 0.46
Model fit					
Log-likelihood	−4242.5836		−4244.3159		
Pseudo R2	0.1795		0.1975		
AIC	8501.167		8502.632		
BIC	8562.051		8556.063		
Observations	14,920		15,260		

^a Significance: p < 0.01.^

^b Significance: p < 0.05.^

### 3.3 LCM results

Prior to LCMs used to analyze preference heterogeneity, it is essential to consider a range of possible class numbers to improve the model’s performance. This study evaluated the fit of models using fit indices. Lower values of BIC and CAIC, as well as higher values of log-likelihood indicate better fit, but it is also important to consider the interpretability and substantive meaning of the classes. In both groups, the model performed better when the number of classes reached 4. Therefore, the four-class model incorporating individuals’ characteristics was chosen (Table 4).

**Table 4:** Model performance with different number of classes.

	COVID-19 vaccine (*N* = 746)				Influenza vaccine (*N* = 763)			
Number of latent classes	2	3	4	5	2	3	4	5
Log-likelihood	−4162.210	−4047.660	−3952.929	−3881.149	−4044.581	−3941.215	−3792.420	−3756.619
CAIC	8538.249	8481.856	8465.101	8494.248	8227.863	8113.597	7908.475	7929.339
BIC	8564.249	8528.856	8533.101	8583.248	8212.863	8088.597	7873.475	7884.339

In the COVID-19 vaccine group, most attributes in the LCM similarly influenced public preferences significantly. However, the impact of attributes varied among the four classes. A total of 35.22% of respondents were assigned to Class^1^ 2, 29.60% to Class^1^ 4, 25.66% to Class^1^ 3, and 9.52% to Class^1^ 1. For Classes^1^ 1, 2, and 4, the sequelae attribute was still the most important attribute, while the out-of-pocket cost became the priority consideration in Class^1^ 3. However, the estimates of most attributes in Class^1^ 3 were similar, indicating this class exhibited unobvious preferences among five attributes. The effectiveness attribute was still the second important one in Classes^1^ 1, 3, and 4. The protection duration produced a more significant effect in Classes^1^ 1 and 2 than Classes^1^ 3 and 4.

According to the class membership, the individual characteristics significantly associated with classes included age, marriage, education, health, residence, yearly household income, vaccination of acquaintances, and risk perception. Class^1^ 1 was less-educated respondents with a relatively high level of income, who were influenced by the vaccination behavior of leaders in their community or workplace, without personally knowing vaccinated doctors, and perceiving a low probability of COVID-19 infection. Class^1^ 2 was younger, unmarried, and healthy adults with low level of income, living in rural areas, acquainted with vaccinated doctors, and perceiving high severity of infection, mild vaccine sequelae, but a high probability of vaccine sequelae. Class^1^ 3 was those unmarried, healthy, and living in rural areas, and they perceived mild vaccine sequelae (Table 5).

**Table 5:** Four classes in the COVID-19 vaccine group.

COVID-19 vaccine (N = 746)								
Class^1^	1		2		3		4	
Share	9.52%		35.22%		25.66%		29.60%	
Attribute	OR	95% CI	OR	95% CI	OR	95% CI	OR	95% CI
Effectiveness (base: 75%)								
95%	2.21^a^	1.87, 2.62	1.60^a^	1.58, 1.62	1.03^a^	1.02, 1.03	1.19^a^	1.08, 1.32
Protection duration (base: 6 months)								
1 year	NS		1.29^a^	1.26, 1.33	0.98^b^	0.97, 0.99	0.90^b^	0.83, 0.98
5 years	1.99^a^	1.32, 3.04	4.07^a^	3.92, 4.23	NS		NS	
Sequelae probability (base: high)								
Middle	2.21^a^	1.68, 2.90	NS		1.02^a^	1.00, 1.02	1.15^a^	1.04, 1.28
Low	4.26^a^	3.05, 5.94	5.21^a^	4.99, 5.43	NS		1.36^a^	1.16, 1.61
Country-of-origin (base: imported)								
Domestic	0.54^a^	0.45, 0.66	NS		NS		0.93^a^	0.90, 0.97
Out-of-pocket cost (CNY) (base:0)								
150	NS		0.39^a^	0.38, 0.40	1.04^a^	1.03, 1.06	0.92^a^	0.88, 0.97
300	NS		0.16^a^	0.16, 0.17	1.03^a^	1.01, 1.05	0.88^a^	0.81, 0.96

Class membership	Coef.	95% CI	Coef.	95% CI	Coef.	95% CI	Baseline	
Age	NS		−0.02^a^	−0.03, −0.01	NS			
Married	NS		0.54^a^	0.20, 0.89	0.63^a^	0.28, 0.97		
Education	−1.70^a^	−2.35, −1.04	NS		NS			
Unhealthy	NS		−0.33^a^	−0.51, −0.16	−0.35^a^	−0.54, −0.16		
Urban regions	NS		−0.28^c^	−0.58, 0.02	−0.84^a^	−1.15, −0.53		
Income (CNY)	0.51^a^	0.24, 0.78	−0.19^a^	−0.29, −0.09	NS			
Vaccinated acquaintances								
Doctors	−1.65^b^	−3.00, −0.30	0.63^a^	0.24, 1.02	0.69^a^	0.28, 1.10		
Leaders	2.11^a^	0.66, 3.57	NS		−0.54^b^	−1.02, 0.10		
COVID-19 infection								
Low probability	0.43^a^	0.05, 0.81	NS		NS			
Mild harm	NS		−0.49^a^	−0.65, −0.32	NS			
Vaccine sequelae								
Low probability	NS		−0.13^b^	−0.25, −0.01	NS			
Mild	NS		0.25^a^	0.13, 0.38	0.14^b^	0.00, 0.28		
Observations	14,920							

Only significant variables were displayed.

Class^1^: Class in the COVID-19 vaccine group.

^a Significance: p < 0.01.^

^b Significance: p < 0.05.^

^c Significance: p < 0.1.^

In the influenza vaccine group, most respondents were assigned to Class^2^ 1, accounting for 34% of those who had identical preferences, ranking of attributes, and similar ORs with their significance to those in the CLM. Similarly, the ranking of the attributes in Class^2^ 3 (22.41%) was the same as that in the CLM, except for that the sequelae were placed before the effectiveness attributes. Class^2^ 2, taking up 27.75%, was the only class placing the most importance on the out-of-pocket attribute, and less attention on the effectiveness attribute, showing great differences from the whole sample. A minority of respondents were assigned to Class^2^ 4 (15.84%), and non-significant estimates of attributes were higher than any other classes.

The estimates of individual characteristics indicated that classes were significantly correlated with age, marriage, education, residence, health, yearly household income, work engagement, vaccination of acquaintances, and risk perception. For Class^2^ 1, respondents were characterized by older age, lower education level, good health, and living in urban regions. They were acquainted with vaccinated doctors and perceived a low probability of infection and a low risk of vaccine sequelae. The individuals in Class^2^ 2 were unmarried and healthy adults without occupations, earning less money, and having vaccinated friends or relatives. They showed a higher perceived risk of influenza infection and a higher perceived probability of influenza vaccine sequelae. Class^2^ 3 was older, unhealthier, had vaccinated friends or relatives, was not acquainted with vaccinated doctors, and perceived a high risk of infection but a low risk of vaccine sequelae (Table 6).

**Table 6:** Four classes in the influenza vaccine group.

Influenza vaccine (*N* = 763)								
Class	1		2		3		4	
Share	34.00%		27.75%		22.41%		15.84%	
Attribute	OR	95% CI	OR	95% CI	OR	95% CI	OR	95% CI
Effectiveness (base:40%)								
70%	1.39^a^	1.27, 1.53	1.07^a^	0.99, 1.15	1.60^a^	1.18, 2.16	NS	
90%	2.14^a^	1.96, 2.34	1.09^a^	1.03, 1.16	5.71^a^	4.08, 8.15	4.44^a^	1.40, 6.04
Protection duration (base: 6 months)								
1 year	1.23^a^	1.14, 1.31	NS		1.96^a^	1.06, 3.63	NS	
Sequelae possibility (base: Middle)								
Low	1.48^a^	1.36, 1.61	1.13^a^	1.07, 1.18	6.72^a^	2.88, 15.64	1.21^a^	0.56, 2.63
Country-of-origin (base: imported products)								
Domestic	1.17^a^	1.11, 1.22	0.88^a^	0.83, 0.93	1.60^a^	1.20, 2.13	0.64^a^	0.14, 2.99
Out-of-pocket cost (CNY) (base:0)								
150	0.56^a^	0.52, 0.61	1.27^a^	1.19, 1.35	0.40^a^	0.22, 0.72	NS	
300	0.37^a^	0.32, 0.43	1.21^a^	1.12, 1.31	0.21^b^	0.05, 0.85	0.77^b^	0.34, 1.72

Class membership	Coef.	95% CI	Coef.	95% CI	Coef.	95% CI	Baseline	
Age	0.04^a^	0.02, 0.06	NS		0.04^a^	0.02, 0.06		
Married	NS		0.36^a^	0.16, 0.57	NS			
Education	−0.20^b^	−0.38, −0.01	NS		NS			
Unhealthy	−0.16^a^	−0.29, −0.03	−0.07^a^	−0.13, −0.02	0.51^a^	0.34, 0.68		
Urban	0.27^a^	0.12, 0.42	NS		NS			
Having a job	NS		−0.11^b^	−0.23, −0.01	NS			
Income (CNY)	NS		−0.75^b^	−1.27, −0.22	NS			
Vaccinated acquaintances								
Doctors	0.71^a^	0.40, 1.01	NS		−0.87^a^	−1.62, −0.13		
Friends/relatives	NS		0.66^a^	−0.01, 1.34	2.87^a^	2.05, 3.68		
Influenza infection								
Low probability	0.43^a^	0.29, 0.58	−0.15^a^	−0.24, −0.06	−0.17^a^	−0.28, −0.06		
Mild harm	NS		−0.21^a^	−0.31, −0.11	−0.40^a^	−0.59, −0.21		
Vaccine sequelae								
Low probability	0.07^b^	0.01, 0.12	−0.37^a^	−0.52, −0.24	0.23^a^	0.12, 0.34		
Mild	0.44^a^	0.26, 0.62	NS		0.44^a^	0.33, 0.56		
Observations	15,260							

Only significant variables were displayed.

Class^2^: Class in the influenza vaccine group.

^a Significance: p < 0.01.^

^b Significance: p < 0.05.^

^c Significance: p < 0.1.^

## 4 Discussion

This study explored the differences and similarities in individuals’ preferences for a newly created vaccine (COVID-19) and a well-established vaccine (influenza) and individuals’ characteristics affecting these preferences. The findings indicate that participants have the same preferences for both types of vaccines, significantly preferring domestic vaccines with higher effectiveness, longer protection duration, a lower probability of vaccine sequelae, and less out-of-pocket cost. Moreover, no matter which vaccine group, effectiveness and sequelae emerged as the most prominent factors in shaping preferences. However, these two attributes differ in two groups. Vaccine sequelae were viewed as more significant than effectiveness when facing the choices of COVID-19 vaccines, whereas the order was reversed for the influenza vaccine.

The finding in the COVID-19 vaccine group is consistent with the research by Antonopoulou et al. (65), Leng et al. (35), and Chan et al. (17), which emphasizes that vaccine safety is predominantly associated with vaccination intentions. However, this contrasts with other studies that list effectiveness as the most essential factor (11, 23, 24). This discrepancy is understandable, as the survey was conducted during a COVID-19 pandemic peak from October to December 2021, a period marked by heightened concerns about vaccine side effects due to extensive media coverage and public discourse. In addition, the sequelae attribute was ranked into three levels in our study, which further enhanced the perceived risk of sequelae.

The results of the influenza group align with most influenza studies (18, 66). The perception that an established vaccine is safer than a fast-paced, developed vaccine contributed to listing sequelae as the first priority (8). Influenza vaccines are often perceived with less uncertainty due to the availability of long-term data. In contrast, COVID-19 vaccines are viewed with greater concern. The differences in risk perception lead to different vaccine preferences and decision-making (8, 9, 52, 67–69).

The preferences incorporated with class membership suggest that populations with varying characteristics exhibit heterogeneous preferences. According to the results for the COVID-19 vaccine subgroup, younger respondents belonging to Class^1^ 2, compared to older samples in other classes, attached more weight to the protection duration. Respondents with different levels of income had distinct attitudes toward the origin and out-of-pocket costs. Class^1^ 1 earning more money preferred imported COVID-19 vaccines and gave little consideration to the cost, while low-earning individuals in Class^1^ 2 considered lower cost as a driver of their preferences and had no significant preferences for the origin of vaccines. Risk perception generated the largest impact on Class^1^ 2. Despite perceiving mild sequelae, high severity of infection and probability of sequelae rendered them to prioritize the vaccine sequelae attribute. For most classes, the vaccination status of acquainted medical experts or leaders was outstandingly significantly correlated with preferences.

Based on the results of the influenza vaccine subsample, Class^2^ 3 was more sensitive to vaccine attributes and easily influenced by risk perception than other classes. Individuals in this class perceived mild risk of the influenza vaccine but prioritized considering the sequelae attribute, which can be explained by their older ages and poorer health. Additionally, age and health may render them at increased risk of virus infection, making the effectiveness attribute the second most important factor. By contrast, the older but healthier respondents in Class^2^ 1 seemed insensitive to each attribute, perceiving a low risk of both infection and sequelae. To our surprise, in Class^2^ 2, out-of-pocket cost was highly emphasized prior to effectiveness and sequelae attributes regardless of a high-risk perception of influenza infection and a perceived high probability of vaccine sequelae, perhaps in that individuals were healthy, not engaged in work, and had a lower level of income. Most class preferences were significantly correlated with the vaccination status of acquainted doctors or friends, as well as risk perception.

To sum up the similarities in both vaccines, lower-income individuals in both vaccine subgroups took out-of-pocket as an important consideration, which is in line with Dong et al.’s (24) finding that the public with lower incomes will pay more attention to vaccine prices. The classes sensitive to effectiveness and sequelae attributes were significantly associated with their risk perception. The more individuals value vaccine efficacy and safety, the higher they perceive risk (44, 48, 49). Health conditions and knowing vaccinated acquaintances in both subgroups were significant for respondents to assess the utility of a vaccine. Being in good health can lessen individuals’ concerns about the benefits and side effects of a vaccine. The impact of vaccinated acquaintances is consistent with previous studies (12, 26, 44, 47).

For differences between subgroups, compared to the COVID-19 vaccine, the impact of the influenza vaccine attributes varied more noticeably across different classes, including the vaccine effectiveness, sequelae, and out-of-pocket cost attributes. As for the inconsistency in the effectiveness and sequelae attributes, the first reason is related to the characteristics of viruses; second, rapidly developing new vaccines can cause a common high-risk perception of vaccines; however, as new vaccines become established, perceived risk will differ among populations. The difference in the cost attribute can be explained by the Chinese context, where COVID-19 vaccines are provided free of charge to citizens, while influenza vaccines require payment. In terms of influence from acquaintances’ vaccination status, doctors played a more important role in shaping COVID-19 vaccine preferences, while influenza vaccine preferences were more impacted by friends or relatives. Plenty of existing studies have found that vaccination decision-making can be impacted by social networks (12, 26, 44, 47). Our findings reaffirm the roles of doctors, friends, families, and influencers and make a further distinction. When it comes to a newly created vaccine, people may assume that medical professionals possess more specialized knowledge and information about the new vaccines, and tend to reference medical experts’ vaccination behaviors. However, for established vaccines, there are more vaccination experiences, so people are more likely to be influenced by friends’ and relatives’ behaviors.

### 4.1 Implications

The results of this study can provide insights into vaccination interventions and policy-making. For newly developed vaccines, such as the COVID-19 vaccines, several strategies can effectively address public concerns. Firstly, given the heightened concern about potential long-term effects of new vaccines, public health campaigns should focus on transparently addressing these concerns. Providing comprehensive information about potential side effects, how to manage them, and the overall safety of the vaccine can help alleviate fears. Secondly, the influence of doctors and medical professionals on vaccine preferences is significant and should be considered. Policies should encourage healthcare providers to actively discuss the benefits and risks of novel vaccines with their patients. Training programs can enhance their ability to communicate effectively about vaccines.

For long-existing vaccines such as the influenza vaccines, highlighting their effectiveness should be prioritized. First, messaging should focus on the benefits of vaccination, especially for older adults and those with poorer health who are more vulnerable to influenza complications. Apart from doctors, friends or relatives also impact vaccine decisions. Public health initiatives should engage these groups, encouraging them to share positive vaccination experiences and information within their networks.

For general strategies, clear and accurate communication about vaccine risks and benefits is essential, particularly during times of heightened public concern, such as pandemic peaks. Especially, to address the specific concerns and needs of unhealthy individuals, it is essential to provide information on the benefits of vaccination for their particular health conditions, potential side effects, and the importance of vaccinations in preventing complications. This includes regularly updating the public on the latest research findings and swiftly addressing misinformation. Moreover, to ensure that cost is not a barrier to vaccine uptake, policies should consider implementing income-based subsidies or free vaccination programs. Finally, mobilizing community leaders, influencers, and vaccinated individuals can help disseminate positive vaccination messages and experiences. Public health campaigns should leverage these trusted voices to increase vaccine confidence and uptake.

## 5 Limitations

There are some limitations to this study, which can be addressed by future research. First, to reduce the burden on respondents, only the five most important attributes were included in the DCEs, excluding factors such as vaccination convenience, sites, and doses, which may also significantly impact vaccine preferences. Similarly, some potentially influential factors, such as government mandates, political movements, news, and personal beliefs in vaccines, were excluded for the same reason. Future studies could incorporate these additional factors for a more comprehensive analysis. Second, despite our efforts to mitigate biases, self-reported data are inherently subject to social desirability and inaccurate reporting biases. Therefore, our findings should be interpreted with caution. Additionally, the DCEs rely on predefined attributes and hypothetical scenarios that are different from the real world, failing to capture the nuances of individual vaccine preferences and additional factors. Future research incorporating qualitative methods, such as interviews or focus groups, could provide a more comprehensive understanding of the factors influencing vaccination decisions. Moreover, the latent class logit model used in this study has limitations in fully capturing the complexity of vaccination decision-making. Future research should explore alternative modeling approaches to provide a deeper understanding of the factors influencing vaccination decisions. Furthermore, our study captured vaccine preferences at a specific time, which might not reflect changes as new information becomes available. Future research can be longitudinal studies to track how preferences evolve. Finally, our study is specific to the Chinese context, these results may not be generalizable to other countries or cultures due to differences in healthcare systems, vaccination experiences, and public perceptions.

## 6 Conclusion

In conclusion, this study aimed to investigate the influencing factors of vaccination decisions for newly developed and established vaccines in China using latent class logit models. Our findings reveal significant similarities and differences in the determinants of vaccination decisions between COVID-19 and influenza vaccines. Key factors include vaccine safety and effectiveness, income, health status, risk perception, and vaccination behaviors of acquaintances. Understanding these factors can help public health authorities design targeted interventions to improve vaccination rates and address vaccine hesitancy. Future research should continue to explore these dynamics in different populations and contexts to further enhance our understanding of vaccination behavior.

## Data Availability

The raw data supporting the conclusions of this article will be made available by the authors, without undue reservation.
