# Corrigendum: Investigating the influencing factors of vaccination decisions for newly-developed and established vaccines: a comparative study based on latent class logit models in China

**DOI:** 10.3389/fpubh.2024.1509718

**Published:** 2024-11-13

**Authors:** Shiyun Chang, Biao Xu, Hailing Xi, Yifan Shao

**Affiliations:** School of Government, Nanjing University, Nanjing, China

**Keywords:** COVID-19 vaccines, influenza vaccines, latent class logit model, choice experiment, vaccine preferences

In the published article, there was an error regarding the corresponding author. The corresponding author was incorrectly assigned as the submitting author. However, the second author should have been designated as the corresponding author. The corrected corresponding author appears below.

^*^Correspondence:

Biao Xu

xubiao@nju.edu.cn

The authors apologize for this error and state that this does not change the scientific conclusions of the article in any way. The original article has been updated.

